# Information and communication technology and climate change adaptation: Evidence from selected mining companies in South Africa

**DOI:** 10.4102/jamba.v8i3.250

**Published:** 2016-04-29

**Authors:** Bartholomew I. Aleke, Godwell Nhamo

**Affiliations:** 1 Department of Agricultural Economics, Management & Extension, Ebonyi State University, Nigeria; 2 Institute for Corporate Citizenship, University of South Africa, South Africa

## Abstract

The mining sector is a significant contributor to the gross domestic product of many global economies. Given the increasing trends in climate-induced disasters and the growing desire to find lasting solutions, information and communication technology (ICT) has been introduced into the climate change adaptation mix. Climate change-induced extreme weather events such as flooding, drought, excessive fog, and cyclones have compounded the environmental challenges faced by the mining sector. This article presents the adoption of ICT innovation as part of the adaptation strategies towards reducing the mining sector’s vulnerability and exposure to climate change disaster risks. Document analysis and systematic literature review were adopted as the methodology. Findings from the study reflect how ICT intervention orchestrated changes in communication patterns which are tailored towards the reduction in climate change vulnerability and exposure. The research concludes with a proposition that ICT intervention must be part of the bigger and ongoing climate change adaptation agenda in the mining sector.

## Introduction and background

The mining industry makes significant contributions to the gross domestic product (GDP) of many countries (Florini 2011). A snapshot on facts and figures shows that in 2011, the mining sector contributed 8.8% of the total GDP in South Africa. Against this backdrop, mining activities are seen as the source of livelihoods for many communities in such countries (Yun-Qiang & Hai-lui 2010). South Africa, to be precise, remains a cornucopia of mineral riches. It is the largest producer of chrome, manganese, and platinum (SANEDI 2013) and also produces significant quantities of coal, gold, diamonds, and iron ore.

Globally, the mining sector is, however, struggling to cope with numerous environmental impacts, especially global warming that manifests in climate change (CC). Climate change-induced disaster risks (DR) and perhaps risks associated with human anthropogenic activities constitute the progenitors of the environmental impacts. ‘Climate change-induced risks probably remain the biggest single threat to the mining industry in the 21^st^century’ (Zhang, Miao & Guo 2009:395). Suffice it to say that the two phenomena, CC and DR, are mutually inclusive in the sense that CC actually orchestrates disaster occurrence. Kramers *et al*. (2014) attribute the increased frequency and severity of natural disaster occurrences to CC. Severe natural disasters affect and disrupt social and economic activities (Appiah & Osman 2014). Highlights of some of the disruptions include heavy floods that disrupted virtually all economic activities in the most affected area of Nigeria in 2012. In 2012, wildfires punctuated economic activities in Perth, Australia for 4 days. Likewise, in 2011 and 2013 respectively, landslides held economic activities of the mining industries to ransom in both Bangladesh and Chile (EM-DAT 2014). The mining sector seems to be vulnerable to CC, and, needless to say, some activities in the mining sector itself, such as extraction and processing, contribute to global warming and CC.

CC-induced extreme weather events such as flooding, cyclones, droughts, fog and mist, hailstorms, wild fires, as well as extreme snow, have added new dimensions to the challenges and risks in the mining sector. Likewise, greenhouse gas emissions pose both environmental and health challenges. This is so, given that many mining infrastructures were not engineered and built with CC in mind. These risks often disrupt mining operations when they occur, and in most cases orchestrate financial losses in the mining business which ultimately affect the GDP. From a cocktail of risks that include physical, reputational, regulatory, financial, and others, the physical risks following extreme weather events remain a key challenge to the mining sector. A number of studies, among them, Mirza (2003) and Kunkel, Pielke and Changnon (1999), have modelled the weather variability over a period of time and noted that the climate is changing and this change is persistent. The changing climate is partly responsible for the risks outlined earlier. Physical risks take the form of power (electricity) blackouts, displacement of people, landslides, and flooding as well as overall financial risks (Hojers, Dreborg & Engstrom 2011). The changing climate further leads to job losses in the economy and at times political and social unrest and instability (Okereke & McDaniels 2012). In most cases rebuilding damaged structures eats deep into budgets, including the national fiscal budget (Appiah & Osman 2014; Hilty, Lohmann & Huang 2011; Hojers *et al.* 2011). Disasters disrupt mining activities as they may lead to both temporary and permanent closures to operations. Fog, mist, and floods, for example, may delay mine product transportation. Heavy rains can result in coal stockpiles getting wet, which means they cannot be transported for power generation (Sapa 2014). Pits may get flooded and equipment drowned as well as getting damaged.

Overall, the impact of CC has negative economic, social, and environmental consequences, particularly if left unchecked. Therefore, this is where new innovative ways that will facilitate daily activities through information and communication technology (ICT) comes in, particularly in the mining sector. Governments and policymakers are working to manage these consequences (risks) as highlighted in Nhamo (2014). Furthermore, in the suggestions, Nhamo mentions public awareness and efficiency in national communication among stakeholders as key strategies for CC adaptation. Getting the public to be aware and more informed about CC is precedence to adapting to CC. Against this backdrop, businesses need to adopt new communication technologies or restructure their existing communication technology to enable them to build the capacity for efficient communication.

This article interrogates the role of ICT innovation as part of the mining sector CC adaptation strategies towards reducing physical risks of mining operations. Evidence from selected mining companies in South Africa is used to support the arguments. The authors propose that ICT innovation is part of the value additions in the mines for capacity development and public awareness campaign required for CC adaptation. Furthermore, ICTs are seen as vital for negotiation and national communication of CC adaptation strategies. The subject is explored through the lenses of ICT readiness, ICT usage, and existence of indigenous knowledge systems in the mines. The authors’ proposal is informed by Bunker and Smith (2009) who argued that ICT adoption should be part of the capacity-building strategies in managing DR, and in our case CC-induced DR. The research findings could inform the mining sector on the potential contribution of ICT innovation towards CC adaptation, and physical risk reduction. In the context of this study, the ICT components considered are not limited to the use of computers (desktop and laptops). Rather the indicators include mobile phones, fixed telephone lines, internet access, fixed broadband subscription, personal computers, and personal digital assistants (PDAs). Therefore where there is the presence of these indicators and they are used, it is assumed that there is ICT compliance (The International Telecommunication Union 2007). This article has four main sections that include: a literature survey on ICT readiness in the CC adaptation context, the methodology, the findings, and a summary of the article.

## Literature survey

This section presents a literature survey of ICT applications in CC adaptation and physical DR reduction. The literature is explored extensively around ICT readiness and communication technology innovation in the mining sector. The literature further presents the mining value chain, reflecting the stages in which mining products such as coal, gold, diamond, platinum, and iron ore undergo before final entrance into the market. The mining value chain is presented to prepare the context for ICT application towards CC adaptation and physical risk reduction at different stages of the value chain. However, as a precursor to the discussion of ICT readiness in this article, ICT is defined in the context of this article as the computer based communication equipment and telecommunication facilities that enable digital exchange of information. Going by this definition, computers, mobile phones, PDAs, and environmental impact assessment systems are included.

The readiness theory suggests that readiness is more dynamic and complex than just preparedness to undertake specific tasks. Drawing an inference from Nhamo (2013), readiness is a concept that should not be confused with ephemeral preparedness. A case of ephemeral preparedness is where businesses may decide to stash computers at different offices without installing some software that is meant for specific tasks or employing people who have the requisite skills. In such a situation, such an organisation may seem to be ICT compliant at face value but in the real assessment they are not. Therefore, such organisations can only be seen as having the intention to undertake such task but in the real sense are not ready for it. According to Nhamo (2013), readiness has wider parameters and indicators that give assurances that a clear structure and platform is well constituted for individual actions.

ICT readiness is part of a more extensive technology-readiness in the mining sector. The broader technology-readiness spectrum emphasises the technologies that enable safety measures, environmental protection, and cost-effectiveness of operations (Melville 2012). Modern design in mining technologies is linked to laser technologies where computers form the basis of activities. ICT usage is the actual deployment of skilled staff and integration of the ICT facilities into the daily operations of the adopting organisation. Indigenous and local knowledge systems (ILKSs) are the information base for a society, which facilitates communication and decision-making. The ILKSs are dynamic and are continually influenced by internal creativity and experimentation as well as by contact with external systems (Flavier, de Jesus & Navarro 1995). This brings ICT into the deep of general technology-readiness strategy in the mining sector. The ultimate goal of ICT innovation in the mining sector is to facilitate communication and its value addition is for safety of operations as these become climate resilient and compatible. When ICT innovation gives an organisation the opportunity to build efficient communication capacity, there is every likelihood that information will circulate at the right time. When information circulates, not only is awareness raised, but it also prepares the minds of people towards eventualities, and such preparation of mind is part of the resilience mechanism. For example, intensive and frequent heavy rainfall in Mpumalanga Province of South Africa resulted in one of Exxaro Resources Limited’s Grootegeluk coal mines being flooded in March 2014 (McKay 2014).

Heat waves are also traceable to both CC and mining activities. Heat waves, according to the Financial Times of 18 January 2011 resulted in the deaths of mining workers in Chile (Roeth & Wokeck 2011). In the examples given herein, stakeholder communication mechanisms are important. Likewise an efficient weather information system is advantageous not to stop the DR from happening, but to help the businesses affected to cope or build resilience. Early warning systems are inevitable in having the mining sector prepare for such eventualities when they take place. Therefore, devising CC adaptation strategies such as ‘increased public awareness’ and ‘efficient communication mechanisms’ seem to be the way forward because physical risks associated with extreme weather events are more frequent and higher in magnitude in recent times (Nhamo 2014). The next section highlights mining value chain and CC adaptation in the mining sector.

**FIGURE 1:**
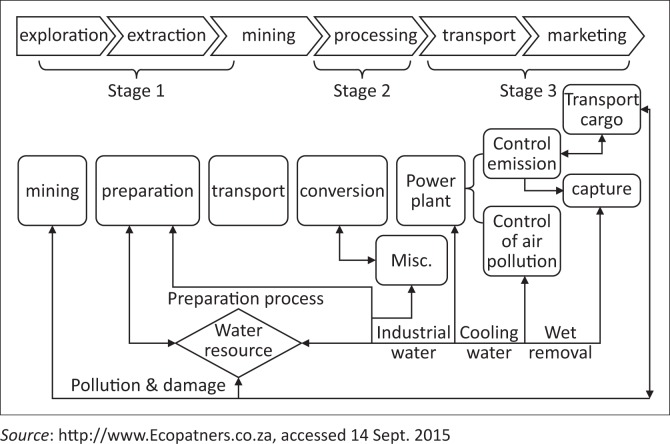
Mining value chain.

It will be an incomplete story to discuss ICT applications in the mining sector without briefly giving the background of the mining value chain. The generic mining value chain embraces the following key stages: exploration, design, extraction, processing, and closure (Vorster 2001). Figure 1 presents the mining value chain.

When tracing the role of ICT in CC adaptation and CC-induced physical risks reduction in the mining sector, it is paramount therefore that one understands the characteristics of such risks across the mining value chain. For example, flooding can impact negatively across the value chain whereas droughts can have a negative impact during processing. This is so because drought initiates water stress and mining companies spend more in sourcing water used both for washing and as the coolant during processing. Fog and mist may have a negative effect on delivery and transportation through destruction of roads to and from mining locations and facilities. Hailstorms may result in electricity blackouts which will severely affect extraction and processing. At every stage of the mining value chain, effective and efficient communication is important either as part of early warning system structure for DR reduction or as a value addition in the mines. Ospina and Heeks (2011:7) rightly put it; ‘efficiency of communication among stakeholders and business networking in the mines’ helps them to build resilience. That is to say, ICT innovation can be harnessed for (1) early warning systems for flooding resulting in timely evacuations, (2) sharing knowledge of adaptation among staff, (3) awareness raising of climate-related risks, (4) coordinating disaster recovery information, (5) supporting consultation and participation in developing adaptation policies, (6) providing training in flood and risks management, (7) providing data to aid adaptation decision-making and (8) gathering and analysing information for vulnerability assessments (Ospina & Heeks 2010; Shabajee *et al.* 2014). A summary in terms of how the mining value chain is affected by the impacts of climate DR is presented in Table 1. The impacts are categorised on a scale low impact (L), medium Impact (M) and high Impact (H).

**TABLE 1:** Impact of climate change-induced events along stages of coal mining value chain.

Disaster/value chain	Exploration			Design			Extraction			Processing			Transportation		
				
	L	M	H	L	M	H	L	M	H	L	M	H	L	M	H
Flooding			√	√					√		√				√
Droughts			√	√				√				√	√		
Fog/mist		√			√			√		√				√	
Wild fires			√			√			√			√			√
Hailstorm		√		√				√			√			√	
Extreme snow			√			√			√		√				√
Heat waves	√			√			√					√	√		
Glacial melting			√		√			√		√				√	
Changes in geographical ranges of flora			√			√			√				√		
Sea level rise	√			√				√		√			√		
Ocean acidification		√		√			√			√			√		

L, low impact; M, medium impact; H, high impact.

Different disaster-orchestrated physical CC risks have different levels of impact along the stages of the mining value chain. Such impacts can be L, M, or H depending on the magnitude and frequency of occurrence of the disaster. The impacts among others include air pollution, unnecessary competition with other small businesses, loss of revenue in the event of disasters and accidents, death, environmental degradation, displacement of communities to make way for mining activities, deforestation, conflicts, and social vices that are associated with mining communities (Appiah & Osman 2014; Hiltey *et al.* 2006; Hojers *et al.* 2011). Various efforts are made to address these impacts. For example, advanced fire information systems (AFIS) is a piece of technology adopted in the Ghana mining sector to checkmate negative impacts of wildfires (AFIS 2015). In some countries, the effort to tame the negative impacts of mining has led to initiating environmental policies geared towards minimising mining’s environmental impact. Therefore, considering the impact of CC in every stage of the mining value chain (exploration, extraction, processing, transporting, and marketing), value protection and addition is required at every stage. For instance, during exploration there are a lot of negotiations and the technology that will facilitate communication among stakeholders is required. Likewise, at the extraction stage, safety information is hugely required, and research and development (R&D) is needed in order to avert physical risks. Similarly, at the transportation stage, there is need to localise weather information in order to re-route distribution channels. At the marketing stage, the mining sector requires market research information on both e-business and global markets to keep abreast of what is happening in other countries.

There is now sufficient evidence that managers of national economies are promulgating CC policies and adaptation strategies to reduce CC-induced DR (Roeth & Wokeck 2011). Such advances by the stakeholders are reflected in Hojers *et al*. (2011) who noted that reasonable effort has been channelled towards institutionalising early warning systems in disaster prone sectors like mining. However, in several instances, the strategies are either still rooted in ILKSs, not timely, or even undermined by laissez-faire attitudes and less goodwill of the stakeholders. Considering the Intergovernmental Panel on Climate Change (IPCC) report of 2012 and the Kyoto protocol, information dissemination is essential in both CC adaptation and response to DR caused by CC. However, in less developed countries, the cost of putting ICT structure in place has always been the bane to good efforts. Drawing from policy positions mentioned in the previous section of this article, clear policies addressing information communication strategies in achieving CC adaptation are nearly non-existent. However, a number of strategies are outlined by Florini (2011) as key climate adaptation strategies in the mining sector. They include the need to: (1) continue developing and improving early warning systems in respect of extreme weather events, (2) facilitate increased uptake of seasonal climate forecast among key stakeholders, (3) maintain and update The Risk And Vulnerability Atlas, (4) investigate and implement plans to use the media and ICT for information sharing, (5) promote R&D in order to explore risk reduction, (6) collaborate with social groups such as community and nongovernmental organisations for awareness and achieving technology transfer and (7) strengthen both formal and informal education with respect to CC, DR reduction, and CC adaptation. Against such backdrop industrial CC adaptation strategies are a reflection of the mining sector CC adaptation policies.

Scholars of high repute, Wastell and White (2013) and Pithouse et al. (2012), have nicknamed ICT intervention ‘change levers’. This is partly because of an efficacy of information sharing on the platform of some ICT components particularly mobile phones. Such effectiveness in information dissemination is required most in the event of a disaster to enable people to evacuate or take necessary precautions. In the mining sector ICT being a change lever, can enhance early warning systems thereby reducing potential risks that at times cause economic losses. However, some practitioners, on the other hand, do not see ICT intervention in a sector as the almighty formula for the sustainability status mentioned earlier. Rather they have accused ICT’s innovation of being an unfriendly august visitor because of havocs done to the environment by giant ICT facility producing companies in a bid to produce ICT gadgets that people use on a daily basis (Aleke, Wainwright & Green 2011). ICT facility producing companies are equally accused of pollution and illicit dumping of waste products, thereby contributing to contamination of the environment that is already under threat of CC-induced disasters (OECD 2009). These parallel arguments have helped us to focus this discussion with respect to identifying risk reduction potentials of ICT intervention in the mining sector. The evidence is pointing out the need to develop further research projects on the correlation between ICT intervention, CC adaptation and DR reduction in other sectors of the economy. The next section discusses the methodology adopted for the study.

## Materials and methods

The article poses the following research question: to what extent is ICT incorporated in addressing CC adaptation by selected South African mining houses? In response to the research question, the primary objective of the study was spelt out as: to determine measures undertaken by selected South African mining houses in incorporating ICT in addressing and enhancing CC adaptation at different stages of the mining value chain. The study gathered data from 10 selected South Africa mining houses through using their annual reports, individual company’s CC policies, carbon disclosure reports, and other relevant documents. The mining houses selected cut across the five top mining subsectors in South Africa that include coal, gold, platinum, diamond, and iron ore.

Table 2 presents the mining houses selected based on five principal products in the mining industry and the documents from each company as well as the province where the corporation is operating in South Africa. A selection of two mines from a designated area was purposive to ensure proper representation and also to take cognisance of the availability of reports and policy statements online. Online availability of relevant documents was part of the selection criteria. Document analysis and systematic literature appraisal were adopted to unravel institutional ICT readiness for DR reduction and CC adaptation in the South African mining sector. This comes against the backdrop of renewed interest in DR reduction as well as shifting emphasis from CC mitigation to CC adaptation and transition to green economy in the mining sector through clean technologies.

Document analysis and systematic literature appraisal have been used extensively in qualitative research (Bowen 2009; Center et al. 2012). Systematic literature appraisal opens up critical discourse of materials studied (Center *et al*. 2012). Similarly, Bowen (2009) maintains that document analysis is well situated in grounded theory more especially from a constructivist point of view. Further in this direction, Maree (2012) maintains that systematic literature survey approach and analyses of publicly available documents are typified, critical, and integrative when using mainly inductive reasoning. Suffice it to say that some advantages bequeath use of document analysis – namely cost-effectiveness of sourcing the materials because of availability in the public domain. Given that not all available online information is authentic, every effort was put into retrieving documents from official selected mining house websites. However, the methodology is not immune to weaknesses, as highlighted by Nhamo (2014). Among the weaknesses pointed out are insufficient details of information and bias of selection. This is partly because there is no laid down protocol for selecting documents to be analysed. These weaknesses of document analysis were ameliorated in this particular study by supplementing CC policy statements with annual reports and Carbon Disclosure Project (CDP) request information reports.

**TABLE 2:** Sample products, mining companies, province and reports.

1	Item	Product	Name of company	Operational province	Documents	Year
1	Gold	Goldfield	Johennesburg-Guateng	Annual report	2013
				Investor CDP information request	2014
2	Gold	Anglogold Ashanti	Free State-Northwest	Annual report	2014
				CDP	2014
3	Platinum	Lonmin Platinum	North-West (Marikane)	Annual report	2014
4	platinum	Anglo-American	Gauteng (Johannesburg)	Annual report	2014
5	Diamond	PetraDiamond	Gauteng (Johannesburg)	Annual report	2014
6	Diamond	Venetia Diamond	Gauteng (Southdale)	Annual report	2014
7	Coal	Anglo-American Thermal	Gauteng (Johannesburg)	Annual report	2014
				Environmental performance standard	2014
				Group climate change policy	2014
8	Coal	Anglo-American Inyosi mine	Mpumalanga	Environmental scoping report	2014
9	Iron ore	Sishen Iron ore	Northern-Cape	Annual report	2010
				Climate change policy	2014
10	Iron ore	Thabzimbi Iron ore	Limpopo	Annual Report	2014
				CDP information request	2014

CDP, Carbon Disclosure Project.

In total 16 documents were analysed comprising nine annual reports, two environmental performance standards, two CC policy statements, and two CDP information reports. This was to ascertain whether ICT intervention is part of their DR and CC adaptation strategies. Using documents as a data gathering technique focuses on all types of written communications that may shed light on the phenomenon that is being investigated (Arts, Fischer & Van der Wal 2012). The documents reviewed were validated to determine whether they followed the protocol of King III^1^ reporting template which requires corporate organisations to include environmental, corporate governance, and social responsibility in their annual reports. The emphasis of the articles is to provide the reflections of such documents about ICT application as part of DR reduction and CC adaptation strategies in the mining sector in South Africa.

## Presentation of results and discussion of findings

**FIGURE 2:**
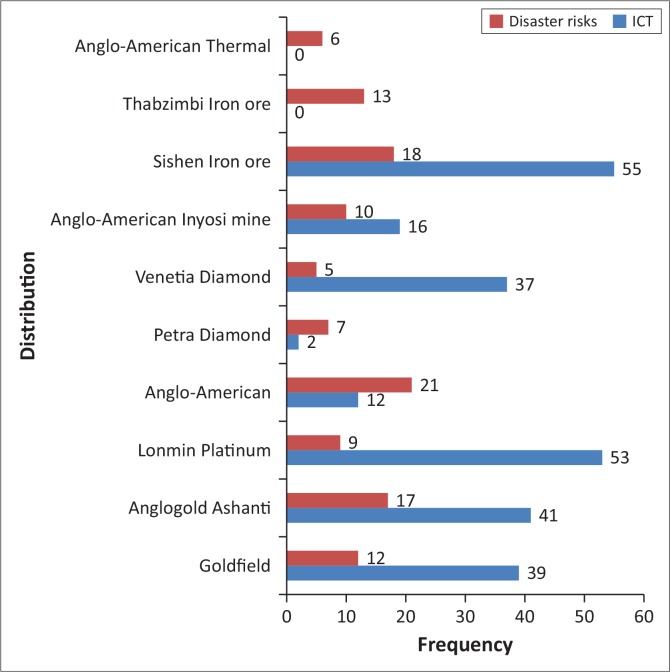
Distribution of mention of information and communication technology and disaster risks in documents analysed.

Following the set research question and objective, the documents were analysed to establish whether ICT interventions reflected in the policy documents or the annual reports as part of CC adaptation strategies or DR reduction approach in the mines. A keyword search using ‘ICT’ and ‘DR’ formed part of the analysis (Figure 2).

Going through the documents to find out whether ICT intervention is part of CC adaptation strategies or part of risk reduction strategies in South African mines, we identified a couple of other existing strategies already in place that are not necessarily linked to ICT intervention. These include: redesigning and modernisation of mining pits and mining to checkmate excess of floods, adjustment of transportation channels to counter floods and hailstorms, and the adoption of energy efficiency plans and swift shift to the alternative renewable energy mix. In 60% of the documents analysed it appears that ICT intervention did not quite reflect as part of CC adaptation strategies. In the Anglo-America Thermal 2014 annual report and Thabazimbi Iron Ore 2014 annual report, there was no mention of ICT intervention at all, whether as part of CC adaptation or DR reduction strategy. This revelation from our analysis raises further concern as to what exactly are the roles of ICT in the South African mining sector.

Wainwright and Waring (2007), opined that ICT intervention in corporate organisations follows two streams of needs, namely: front-office application and back-office applications. Front-office application talks of ICT readiness in respect to an exchange of information between the group and the outside society. Typical examples of front-office ICT applications include electronic mail communication with stakeholders, electronic advertorials, social media, and electronic billboards. Whereas, back-office ICT applications include payroll service systems, financial bookkeeping systems, and database management systems. Suffice it to say that ICT can be useful in many fabrics of organisational value chain especially, in networking with other businesses. However, the objective of this study is within the scope that is focused on ICT intervention and DR reduction.

The missing out of ICT intervention in the CC policy documents of many mining companies raises a suspicion of ICT intervention being skewed towards back-office applications instead of being part of CC adaptation strategy or DR reduction mechanism. Even though, ICT intervention was not built into the mainstream of international CC adaptation policies (IPCC 2012; UNFCCC 2007) the CC adaptation policy of Anglo-Ashanti mine, one of the documents analysed, highlights the options available to individual organisations to localise their CC adaptation strategies and also DR reduction framework. Against this backdrop, individual mining companies in South Africa have the option to integrate ICT intervention in their CC adaptation and DR reduction blueprint (master plan). Apart from this, the Department of Environmental Affairs (DEA 2011) insisted that businesses in South Africa should target ‘smart environment’ in every one of their business operations. Smart environment according to the DEA (2011) master plan is where risk factors to both people and business are reduced to barest minimum and safety is guaranteed. Smart environment also encompasses different kinds of smart devices continuously working to make inhabitants’ lives more comfortable. The dual emphasis on CC adaptation and reduction in physical risks is reflected in Goldfield (2014) annual report that proposes building community resilience to CC through network formation and environmental sustainability as a prerequisite for smart society. An extract from the document under analysis is presented in Box 1.

BOX 1 Goldfields on climate change and information dissemination.

“The most important intervention in Gold Fields’ long term (more than 3 years from now) strategy, influenced by climate change has been the formal incorporation of climate change considerations into the process of developing new mining operations. This has been supported by the development of guidelines to support the integration of mitigation and adaptation-related issues into asset design. This will start from supporting mining hosting communities to build resilience. We will support networks that will make information reach the right people. Again Gold Fields’ development teams are required to calculate the new asset’s carbon footprint over its lifetime and to identify energy efficiency and renewable energy opportunities early in the development process. Furthermore, Gold Fields has set a target that all new mining projects must at least have 20% of their energy sourced from alternative sources of energy”.

*Source*: Goldfield, 2014, Integrated Annual Report (IAR) (2014) Prepared for Goldfield South Africa, viewed 12 September 2015, from http://www.goldfields.com

Sustainability cuts across several other documents analysed. The statement in Box 1 portrays clear awareness of the impact of CC and its associated DR in the mining business. It also points out commitments towards CC adaptation and DR reduction. Likewise, in Anglo-American Thermal Environmental Performance Standard 2014 annual report, Anglo-American Inyosi Mine Environmental Scoping Report 2014, and Venetia Diamond 2014 annual report, sustainability was reflected to cover broad subjects which include water management and social sustainability. The particular interest of this article is vested on the social sustainability that covers indicators such as reduced harm to people, awareness generation, improvement in education, improvement in ICT, and social inclusion. Social inclusion on its own is an interesting theory because it borders on gender, care to the elderly, vulnerable children, physically challenged human beings, and the economically vulnerable in the society. The reflection here goes back to the research question, exploring the paramount role of ICT intervention in achieving these sustainability indicators mentioned. The next section highlights vulnerability of mining sector to CC impact and DR.

CC documents of the majority of mining companies studied openly acknowledged that CC is a significant threat to mining activities, and adaptation strategies require urgent implementation. CC policies of Anglo-America Thermal (2014) explicitly state the need to increase the financial allocation to CC adaptation and energy use because of what the document regarded as ‘uncertainties’. Similarly, Sishen Iron Ore CC policy 2014, which is incorporated in the 2014 annual report, included DR reduction in their mission statement because of what the document regarded as a severe impact of extreme weather conditions. An extract from the Anglo American plc 2012 Annual Report addressing climate change risk is shown in Box 2.

BOX 2 Anglo American plc addressing climate risk.

Some of our operations are located in areas exposed to natural catastrophes such as earthquake/extreme weather conditions. The impact of climate change may intensify the severity of weather events. The nature of our operations exposes us to potential failure of mining pit slopes and tailings dam walls, fire, explosion and breakdown of critical machinery, with long lead times for replacement. Specialist consultants are engaged to analyse such event risks on a rotational basis and provide recommendations for management action in order to prevent or limit the effects of such a loss.

Contingency plans are developed to respond to significant events and restore normal levels of business activity. Anglo American purchases insurance to protect itself against the financial consequences of an event, subject to availability and cost.

*Source*: Anglo American plc, 2012, *Anglo American plc Annual Report 2012*, viewed 16 April 2016, from http://www.angloamerican.com/~/media/Files/A/Anglo-American-Plc/arsdr2013/AR_p52.pdf

Further in the findings, we identified that the mining sector is not only vulnerable to CC physical risks. The mining sector is equally vulnerable to the adverse impact of its waste product, especially the mine sewage. Therefore there is need to raise awareness of such impact as part of value protection in the mining sector. Bunker and Smith (2009) equally acknowledged the challenging situation does not translate to a solution until a practical step is adopted towards creating a solution. Against this backdrop, ICT intervention can play a huge role in constituting early warning systems at the exploration and mining stages where floods and hailstorms can be problematic in the mining value chain. Geographical information systems (GIS) and remote sensing technologies can generate valuable information required during negotiation for operating licences and community liaisons. Indeed, the vulnerability of the mining sector to CC and its associated DR in a natural resource and agricultural dependent country like South Africa goes beyond the boundaries of the stages of the value chain. Neither does it stop at the economic loss statistics shown in the company’s annual balance sheet. The multiplier effects of these financial losses, as a result of disasters, are reflected more on the livelihood of the people within the mining communities.

**FIGURE 3:**
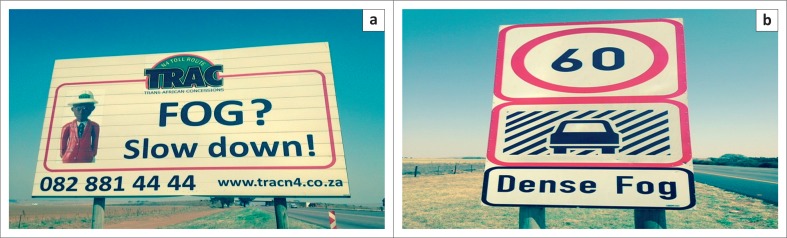
Fog sign (a) along the N4 road and a heavy fog (b) sing along the N4 road in Mpumalanga Province.

Gathering information on fog and mist and putting it into a mine’s communications strategy and/or GIS may add value as a CC adaptation strategy. This could be linked to a live driver update through social media platforms like WhatsApp. Driving across the mining hub of South Africa, thus, the Mpumalanga Province roads are dressed with fog signs that the mining sector can utilise to build GIS and communications strategies for CC adaptation. Such a GIS and communications strategy could be enriched by searching historical data on the number of days in which such events occur annually from the official weather services departments. Figures 3a and b show captions in terms of road signs warning of fog and heavy fog in Mpumalanga Province of South Africa.

The Lonmin Platinum 2014 annual report contains a section that speaks directly to corporate social responsibilities of the mines to the communities where women, youths, elderly, and children are most affected by CC-induced disasters and risks associated with anthropogenic mining activities. Part of the social responsibilities captured here include CC awareness programmes, gender balanced formal education, bursaries and royalties, improvement in ICT, and local content recognition in employment.

DR reduction emerges as a guided principle of CC change policies and actions. Admittedly, not only that the mining companies surveyed want to reduce environmentally associated risks, but also wish to keep every other risk such as financial risks involved in doing business down. The enabling pillars for DR reduction were identified from the documents analysed. These include, but are not limited to increase in budget (finance), knowledge management, capacity building, technology transfer, and infrastructure upgrade, as well as data management. The data management entails archiving and easy retrieval of climate data for CC adaptation purposes. The latter is achieved through installation of appropriate computer hardware and software. It is therefore not surprising when Kramers *et al*. (2014) suggested that mining industries can leverage on ICT when addressing impacts of CC.

## Conclusion

Part of circumspective evidence of ICT readiness in an organisation or an economic sector that works on a platform of environment is when the system analysts are preoccupied with available environmental simulation and modelling that enables them to localise CC adaptation strategies and develop rapid response frameworks for risk aversion or reduction. Mirroring our findings, one would comfortably argue that ICT intervention in the mining sector has two areas of projection for risk reduction. Firstly is a just-in-time red alert warning, although efficient telecommunication facilities are also needed to be in place for maximum output. Secondly, in reducing health or economic risk one would suggest that mines will transform their indigenous knowledge into digital electronic platforms such as electronic signposts, advertorials, pictures, and flyers for faster and easier information sharing. This venture will enable proactive discovery of what would have led DR to both worker and the company.

We identified the need for system improvement when we matched ICT intervention, CC adaptation strategies, and DR reduction at varying stages of the mining value chain. Starting from exploration to marketing as we went through the documents, the need was evident. In clear terms, adoption of ICT innovation in the mining sector will be appreciated if it will reduce disaster physical risks, reduce the cost of operation, and trigger profitability. Adoption of ICT innovation will also be appreciated in the mining sector if its value addition prospect will facilitate environmental and social sustainability. At the marketing stage of mining value chain value addition is necessary to increase revenue generation of the mines and possibly enhance attraction to investors. At the processing stage, system improvement is required to ensure resource conservation, reduction in pollution, promotion of the use of alternative energy, and innovative technologies. System improvement here sets a good precedence for green transition and not only CC adaptation or DR reduction.

This article contributes to the overall CC adaptation policy framework by creating a scenario for understanding of the dimension of ICTs in CC adaptation and DR reduction. It has captured and presented the effectiveness and role of ICTs in reducing DR at different stages of the mining value chain. It has brought to bay, the contributions of ICTs towards achieving economic, environmental, and social viabilities in the mining sector because ICTs can support public awareness of CC through social media platforms, thereby paving the way for resilience building. This article has opened up areas of further research on the relevance of ICTs in other mining sectors by proposing ICTs adoption for both human capital development and business process re-engineering, especially if the adoption of such ICTs innovation will serve as both value protection and value addition mechanism.

## Footnotes

1 King iii reporting requires integrated representation of a company’s performance in terms of both financial and other value relevant information. Companies are meant to provide greater context for performance data in their integrated annual or quarterly report. They should clarify how value relevant information fits into their operations or business goals, The King iii report of every company should also contain details of social responsibilities of such companies to the hosting communities as well as the companies’ financial statement.
